# Therapeutic effects of internal fixation with support plates and cannulated screws via the posterolateral approach on supination external rotation stage IV ankle fracture

**DOI:** 10.12669/pjms.36.3.1671

**Published:** 2020

**Authors:** Zhongbing Liu, Genling Tang, Shuguang Guo, Bin Cai, Qingsong Li

**Affiliations:** 1 Zhongbing Liu, Department of Orthopedics, Affiliated Taizhou People’s Hospital of Nantong University, Taizhou 225300, Jiangsu Province, P. R. China; 2 Genling Tang, Department of Orthopedics, Affiliated Taizhou People’s Hospital of Nantong University, Taizhou 225300, Jiangsu Province, P. R. China; 3 Shuguang Guo, Department of Orthopedics, Affiliated Taizhou People’s Hospital of Nantong University, Taizhou 225300, Jiangsu Province, P. R. China; 4 Bin Cai, Department of Orthopedics, Affiliated Taizhou People’s Hospital of Nantong University, Taizhou 225300, Jiangsu Province, P. R. China; 5 Qingsong Li, Department of Orthopedics, Affiliated Taizhou People’s Hospital of Nantong University, Taizhou 225300, Jiangsu Province, P. R. China

**Keywords:** Cannulated screw, Stage IV ankle fracture, Supination external rotation, Support plate

## Abstract

**Objective:**

To evaluate the therapeutic effects of internal fixation with support plates and cannulated screws via the posterolateral approach on supination external rotation stage IV ankle fracture.

**Methods:**

Eighty-five patients with SER-IV° ankle fracture and large posterior malleolar fracture treated from June 2016 to June 2018 in our hospital, were randomly divided into a support plate group (n=47) and a cannulated screw group (n=38). The treatment outcomes were compared regarding surgical time, amount of bleeding, time of fracture healing, postoperative complications, as well as the American Orthopedic Foot and Ankle Society (AOFAS) Ankle-Hindfoot Score and excellent rate one year later.

**Results:**

The surgical time and intraoperative blood loss of cannulated screw group were significantly lower than those of support plate group (P<0.05). There were four cases of posterior lateral incision redness complicated with obvious bloody exudation in support plate group on the postoperative 2nd day. One case developed into superficial incision infection subsequently, and one case suffered from deep infection. After dressing and treatment with sensitive antibiotics, stitch removal was delayed, and primary healing was obtained. In cannulated screw group, there were two cases of posterior lateral incision redness complicated with obvious bloody exudation on the postoperative 3rd day, without skin incision infection. One case had cannulated screw loosening two months after surgery, and the posterior malleolar fracture block was slightly displaced. The incidence of surgical complications in support plate and cannulated screw groups were 8.51% and 7.89%, respectively (P>0.05). The AOFAS scores of cannulated screw ((81.71 ± 12.39) points) and support plate groups ((86.62 ± 10.12) points) were significantly different (P<0.05).

**Conclusion:**

For patients with posterior malleolar fracture or osteoporosis, fixation using support plate is recommended. Cannulated screw fixation is suitable for for patients with poor conditions of skin soft tissues or basic diseases such as diabetes intolerant to long surgery.

## INTRODUCTION

Ankle fracture mostly occurs between 20 and 65 years old, with an annual incidence of about 1/1,000. The posterior malleolar fracture is an important part of ankle fracture, accounting for 7%~44%.^1^ According to the Lauge-Hansen classification, supination external rotation is the most common in ankle fracture, of which stage IV fracture often involves posterior malleolar fracture. The criteria for surgical treatment of posterior malleolar fracture are generally based on the size of fracture block, degree of displacement, stability of ankle joint and ligament injury. Due to wide clinical application of CT imaging, it is generally considered that surgical treatment should be performed for posterior malleolar fracture with an anteroposterior diameter exceeding that of the tibialis articular surface by 25%, and a displacement of over 2 mm. Anatomically, the posterior malleolus, as a stop point for the distal tibiofibular joint with opisthodetic ligament, participating in maintaining the ligament stability. Posterior malleolus fixation is traditionally performed by percutaneously fixing using screws backwards. Although it is minimally invasive, it is difficult to achieve anatomical reduction because of the inability to directly observe the posterior malleolar fracture block, leading to poor prognosis.^2^ Recently, the posterior lateral approach of support plate or cannulated screw has been gradually employed to treat posterior malleolus fractures.^3^ This study retrospectively analyzed 85 patients who underwent internal fixation with support plates and cannulated screws via the posterolateral approach to treat supination external rotation stage IV ankle fracture. The findings are of great significance to future clinical therapies.

## METHODS

### Baseline clinical data

Eighty-five patients with SER-IV° ankle fracture and large posterior malleolar fracture who were treated in our hospital from June 2016 to June 2018 were enrolled as subjects after the approval of Institutional Review Board on June 3, 2016.

### Inclusion criteria:

**Fig.1:**
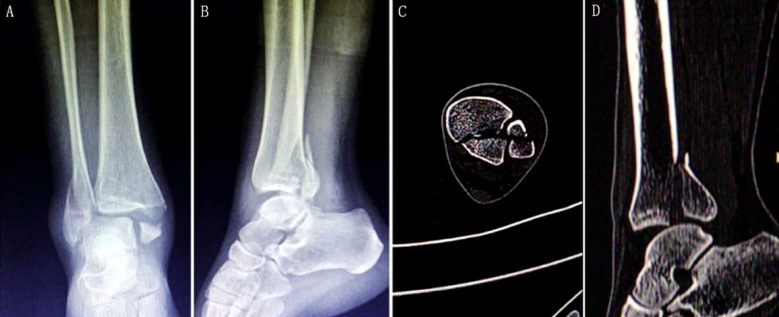
Imaging results a 47-year-old female patient who suffered from 3.5 h of car accident-induced right ankle swelling and pain complicated with limitation of motion before surgery. A: Frontal X-ray film of ankle joint; B: lateral X-ray film; C: CT scan cross-section image; D: CT scan sagittal image.

1. Unilateral fresh closed fracture.
2. Ankle fracture with Lauge-Hansen stage IV, and posterior malleolar fracture involving ≥25% of the tibialis articular surface, with the displacement over 2 mm (Fig.1).
3. All surgeries were conducted by associate chief physicians or above using apparatus from the same manufacturer.
4. With complete follow-up data.

### Exclusion criteria:

1. With skin soft tissue damage or open fracture.
2. With infected wounds on the skin and soft tissue in affected limbs.
3. Medial malleolus extension type of posterior malleolar fracture or posterior malleolus fracture block involving the tibialis articular surface of <25% according to the anteroposterior and lateral X-ray of ankle joint and CT plain scan.
4. Ankle joint dysfunction before injury, or complicated with bone diseases around the ankle, Parkinson’s disease, poliomyelitis, etc.
5. With history of tumor or other immunodeficiencies, blood diseases, and physical conditions that cannot tolerate surgery.
6. With pathological or posterior pilon fractures.
7. With multiple fractures or neurovascular injuries affecting the functional exercise of involved limbs.

The 85 patients consisted of 48 males and 37 females aged 21~66 years old, (42.92±12.26) on average. They were followed up for 12~16 months, with the average of (13.11±1.12) months. There were 32 left and 53 right cases. As to the causes for fractures, there were 36 cases of car accident injury, 26 cases of sprain, 16 cases of fall injury and seven other cases. They were randomly divided into a support plate group (n=47) and a cannulated screw group (n=38) according to the fixation method. The support plate group comprised 29 males and 18 females aged (43.54±12.59) years old. The cannulated screw group consisted of 19 males and 19 females aged (42.21±12.12) years old. The two groups had comparable gender ratio, age, left and right fractures, causes for injury, diabetes, and calcaneal traction before surgery (P>0.05) (Table-I). This study has been approved by the ethics committee of our hospital (dated June 3, 2016), and written informed consent has been obtained from all patients.

**Table-I:** Baseline clinical data of two groups.

	Support plate group (n=47)	Cannulated screw group (n=38)	t/χ^2^	*P*
Age (year)	43.54±12.59	42.21±12.12	0.492	0.624
*Gender*			1.171	0.279
Male	29	19		
Female	18	19		
*Side*			0.346	0.557
Left	19	13		
Right	28	25		
*Cause for injury*			0.039	0.998
Car accident injury	20	16		
Sprain	14	12		
Fall injury	9	7		
Others	4	3		
Diabetes	8	5	0.242	0.623
Preoperative calcaneal traction	7	4	0.356	0.551

### Preoperative preparation

All patients were treated with external plastering after the hospital was placed in the hospital. Those with dislocation were given emergency treatment for manual reduction, which was then fixed with cast. The patients with severe dislocation were performed with calcaneal traction. Routine treatments such as pain relief, stomach protection and anticoagulation were given preoperatively. If the swelling was severe, the appropriate amount of mannitol or glycerin fructose should be applied. For the patients with basic diseases, the relevant indicators should be monitored, and if necessary, the relevant department should be asked for consultation. When tension blisters appeared, the vesicle fluid was removed by a syringe, and the blister skin was retained. After the swelling of the ankle joint subsided, surgical treatment was performed when its surrounding skin showed positive dermatoglyphic characteristics. The time from injury to surgery was 4~10 days, (6.55±1.71) on average.

### Surgical position and approach

The floating position was selected for surgery:

1. The patient was taken a lateral position on the healthy side, with cushions placed under the axillary fossa and the caput fibulae on the healthy side, and the upper limbs were placed on the support plate.
2. The anterior and posterior pelvic fixation frames were respectively placed at the lower back and the symphysis pubis, padded with a cushion and left with a certain gap. The fixation frames were adjusted during surgery to achieve a floating state. The posterior lateral approach combined with the medial approach was selected as the surgical route.

### Surgical method for cannulated screw group

**Fig.2:**
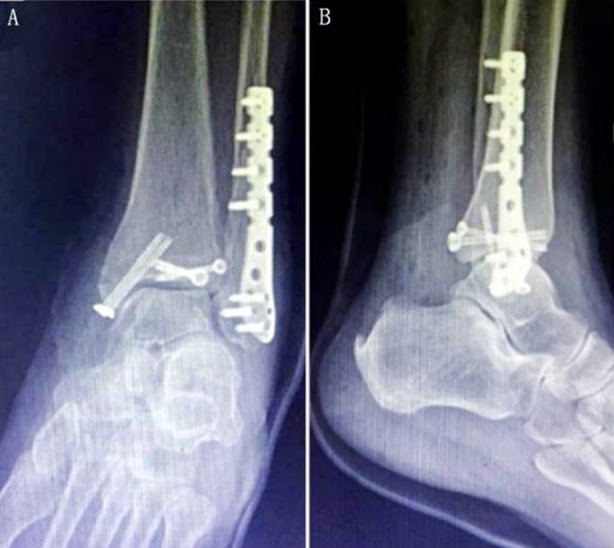
Surgical method for cannulated screw group. A: Postoperative frontal X-ray film of ankle joint; B: lateral X-ray film.

Continuous epidural anesthesia or general anesthesia was chosen. First, the floating lateral position on the healthy side was taken, with balloon tourniquet on the proximal thigh and routine towel disinfection. An incision of 8~12 cm was made along the posterior border of the tibia and the outer midline of the Achilles tendon. The incision started from the end level of the fibula and then extended proximally. The small saphenous vein and the sural nerve were generally located in front of the incision, and the posterior-lateral position of the lateral malleolus, on which attention should be given to protection. The deep fascia was cut open to expose the long and short tendon of the fibula which was retracted backward to expose the broken end of the external malleolus fracture, and to clean the soft tissue and broken bone embedded in the fracture end. Reduction of fracture was performed under direct vision, which was temporarily fixed with towel forceps. For oblique or spiral fibula fracture, a lag screw of a suitable length was selected to fix the vertical fracture line, then a plate of appropriate length was chosen to be placed on the posterior-lateral fibula; then holes were drilled to measure the depth and screwed into the screws of corresponding lengths. Then an incision was made along the starting point of muscle on the outer edge of flexor pollicis longus, which was pulled to the medial side. The posterior malleolar fracture block was exposed, the periosteum was appropriately peeled off, and the soft tissue of the fracture end was cleaned; the fracture was restored under direct vision, and temporarily fixed by two guide needles. During surgery, C-arm X-ray fluoroscopy showed anatomic reduction on tibialis articular surfaces. Then the holes were drilled in turn and two cannulated screws (4.0 mm) were screwed. The position was changed to be supine, and the arc-shaped incision of the facies anterior of medial malleolus was taken to fully expose the fracture end of the medial malleolus fracture, and the soft tissue of the fracture end was cleaned. The fracture was restored under direct vision, and temporarily fixed by two guide needles. The intraoperative C-arm X-ray fluoroscopy revealed the reduction was good, and two cannulated screws of suitable length were screwed after drilling in turn to measure the depth. The Cotton test was performed to examine the distal tibiofibular stability. The instable part was fixed by 3-layer infracortical distal tibiofibular screw about 30° to the inclined forward inside of the lateral malleolus. Then C-arm X-ray fluoroscopy was carried out to find a good reduction, the incision was flushed, and the hemostasis was fully stopped. A negative pressure drainage tube was placed into the posterolateral incision which was performed with tension-reduced suture layer by layer (Fig.2).

### Surgical method for support plate group

**Fig.3:**
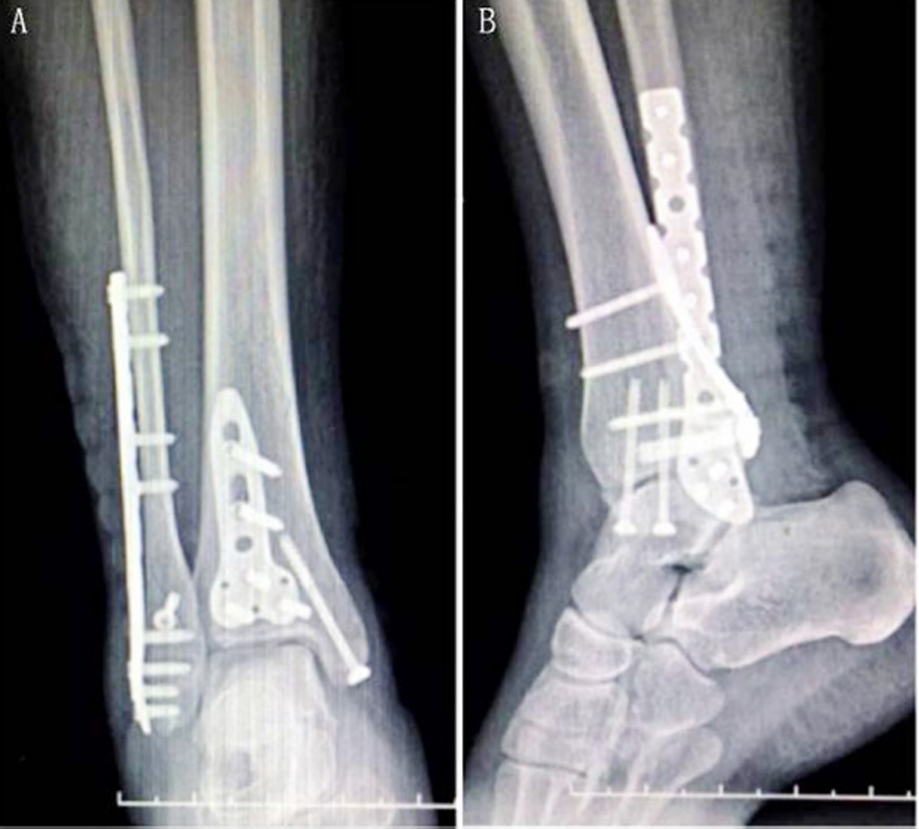
Surgical method for support plate group. A: Postoperative frontal X-ray film of ankle joint; B: lateral X-ray film.

The fixation method of external malleolus fracture was the same as that of the cannulated screw group. An incision was made along the starting point of muscle on the outer edge of flexor pollicis longus, extended to the proximal side, and then pulled to the medial side. The peroneus longus and brevis muscles were pulled to the outside to expose the posterior malleolar fracture block, which was peeled off to the proximal end to clean the soft tissue of the fracture end. The fracture was restored under direct vision, and the point-shaped reduction forceps were temporarily fixed. During surgery, C-arm X-ray fluoroscopy showed anatomic reduction on tibialis articular surfaces. The support plate of an appropriate size was selected, and screws of the corresponding length were sequentially screwed. The remaining surgical methods were the same as those of the cannulated screw group (Fig.3).

### Observation indices and functional assessment

The time from injury to surgery, the surgery time, the intraoperative blood loss, and the fracture-to-bone healing time (X-ray reexamination showed that the fracture line was blurred with successive bone callus) of the two groups were recorded. According to the Ankle-Hindfoot Scoring Criteria of the American Orthopedic Foot and Ankle Society (AOFAS),^4^ the ankle joint function, locomotor activity, pain and maximum walking distance of the affected limb one year after surgery were evaluated, with a total score of 100 points. Then the rate of excellent and good cases to total ones was recorded. 90~100 points: Excellent; 75~89 points: good; 50~74 points: mediocre; <50 points: poor.

### Statistical analysis

All data were analyzed by SPSS16.0 software. Numerical data, such as number of using distal tibiofibular screws and that of cases with postoperative complications, were expressed as frequency or percentage. Intergroup comparisons were performed by the χ^2^ test. Categorical data, such as intraoperative blood loss and surgical time, were represented as mean ± standard deviation (x±s). The variance homogeneity was examined, and the data were compared with the t test. P<0.05 was considered statistically significant.

## RESULTS

There were no significant differences between surgical time, fracture healing time and number of distal tibiofibular screws (P>0.05). The surgical time and intraoperative blood loss of the cannulated screw group were significantly lower than those of the support plate group (P<0.05) (Table-II).

**Table-II:** Surgical time, intraoperative blood loss and fracture healing time.

	Support plate group (n=47)	Cannulated screw group (n=38)	t/χ^2^	*P*
Time from fracture to surgery (day)	6.63±1.82	6.50±1.59	0.346	0.730
Surgical time (min)	139.29±13.78	131.27±13.46	2.696	0.008
Intraoperative loss (mL)	145.79±15.11	125.48±15.48	6.094	<0.001
Fracture healing time (week)	11.67±1.12	11.85±1.13	0.734	0.465
Use of distal tibiofibular screw (case)	5	5	0.129	0.720

There were 4 cases of posterior lateral incision redness complicated with obvious bloody exudation in the support plate group on the 2nd day after surgery. One case developed into superficial incision infection subsequently, and one case suffered from deep infection. After dressing and treatment with sensitive antibiotics, stitch removal was delayed, and primary healing was obtained. In the cannulated screw group, there were two cases of posterior lateral incision redness complicated with obvious bloody exudation on the 3rd day after surgery, without skin incision infection. One case had cannulated screw loosening two months after surgery, and the posterior malleolar fracture block was slightly displaced. The incidence rate of surgical complications was 8.51% in the support plate group and 7.89% in the cannulated screw group (P>0.05).

The AOFAS score of ankle joint one year after surgery in the cannulated screw group was (81.71 ± 12.39) points, including 14 excellent cases, 16 good cases, 6 mediocre cases and 2 poor cases. The rate of excellent and good cases to total ones was 78.95%. The support plate group had a score of (86.62 ± 10.12) points, including 17 excellent cases, 21 good cases, 6 mediocre cases and 3 poor cases. The rate of excellent and good cases to total ones was 80.85%. Their AOFAS scores were significantly different (P<0.05) (Table-III).

**Table-III:** AOFAS score of ankle joint and excellent rate.

	Scoring criteria (case)				Rate of excellent and good cases to total ones (%)	AOFAS score (point)

	Excellent	Good	Mediocre	Poor		
Support plate group (n=47)	17	21	6	3	80.85	86.62±10.12
Cannulated screw group (n=38)	14	16	6	2	78.95	81.71±12.39
t/χ^2^					0.048	2.012
P					0.827	0.024

## DISCUSSION

Recently, Fitzpatrick et al. reported that posterior malleolus fixation improved the stability of the lateral ligament of ankle joint.^5^ Besides, Verhage et al. systematically reviewed articles concerning posterior malleolus fixation for fractures in databases (PubMed, Embase, Cochrane) of the past 22 years^6^, and concluded that preoperative ankle dislocation was an important cause for traumatic arthritis and poor prognosis. Tosun et al. retrospectively analyzed 49 patients with three-ankle fractures and found that the AOFAS score of the unfixed group was significantly lower than that of the mixed group, suggesting that posterior malleolus fixation had a positive impact on the function and prognosis of ankle joint.^7^ In addition, they reported that support plate fixation may no longer need cannulated screw and posterior malleolar fracture required fixation regardless of the size. In this study, there was no significant difference between the number of distal tibiofibular screws (P>0.05). Probably, the operational stability along one direction did not mean that all directions were stable. Rational use of distal tibiofibular screws maintained the ankle mortise stability and prevented the talus from moving outward.

Thus, the size and shape of posterior malleolar fracture were equally important. Through CT imaging, Evers et al. found that the overall prognosis of posterior malleolar fracture with an articular surface involvement of >25% was the best.^8^ Odak et al. pointed out that the final prognosis of three-ankle fracture was only moderate.^9^ Although the size of posterior malleolar fracture was not significantly related to the occurrence of arthritis, prognosis was significantly affected by initial fracture displacement, ankle dislocation, uncoordinated residual joints and talus subluxation.

To achieve anatomical reduction, the floating position was selected for surgery herein. A posterior lateral incision was made to expose external and posterior malleolar fractures, and a satisfactory surgical field of vision was provided to clean the hematoma and soft tissues between fractures. Reduction and fixation were conducted under direct vision, so the placement of posterolateral plate under muscle had biological advantages such as low incidence rates of complications, soft tissue injury and infection. Similar results have been reported by Forberger et al. analyzing 45 patients treated with posterolateral approach^10^ and Choi et al. following up 50 patients.^11^ Nevertheless, the sural nerve should be protected during surgery.

Herein, the surgical time and intraoperative blood loss of the support plate group were significantly higher than those of the cannulated screw group. In terms of postoperative complications, neither group suffered from incision rupture, delayed fracture healing or bon non-union. There were 2 cases of postoperative incision infection in the support plate group, and primary healing was achieved by dressing and antibiotic treatment. We postulated that the support plate group had longer surgical time, more disconnection of soft tissues and larger trauma. In contrast, the cannulated screw group had smaller trauma, less disconnection of soft tissues, simpler surgical procedure and shorter surgical time, so incision infection did not occur. Although the two groups had similar times of bone healing, one case had cannulated screw loosening two months after surgery, and the posterior malleolar fracture block was slightly displaced. One year after surgery, the AOFAS score and the excellent rate of the cannulated screw group were lower than those of the support plate group. Accordingly, the long-term fixation effects of cannulated screw were inferior to those of support plate, which may be related to the small contact area for fixation and the vertical shear force between fracture blocks. Moreover, the one case of screw loosening was 66 years old, probably due to osteoporosis. Differently, Erdem et al. reported that cannulated screw and support plate led to the same prognosis.^12^ As to the difference, we held that two cannulated screws only gave stability along a line, and three screws that formed a plane may work better. Cift et al. concluded that fixation using support plate in combination with screw was stable than screw fixation alone.^13^ It is well-accepted that the fixation strength of support plate exceeds that of cannulated screw. However, Wang et al. reported that for Haraguchi Type-I posterior malleolar fracture, the fixation strength of support plate was lower than that of screw.^14^ Hence, the fixed strength is affected by the size and shape of posterior malleolar fracture block, the direction of fracture line and patients’ own conditions. To clarify the current status of treating posterior malleolar fractures, Gardner et al. conducted a questionnaire survey for members of AOFAS. A total of 401 specialists completed the questionnaire, of whom 56% preferred the posterior lateral approach for internal plate fixation.^15^

## CONCLUSION

In summary, for patients with SER-IV° ankle fracture and large posterior malleolar fracture, fixation using support plate via the posterolateral approach is more beneficial for long-term functional recovery, regardless of longer surgical time and more intraoperative blood loss than those using cannulated screw. Support plate fixation is recommended for patients with posterior malleolar fracture comminution or osteoporosis, it is recommended to choose plate fixation, while cannulated screw fixation is suitable for patients with poor conditions of skin soft tissues or basic diseases such as diabetes intolerant to long surgery.

### Authors’ contributions:

**GT, SG, BC & QL** performed this study, analyzed clinical data and drafted this manuscript.

**ZL** designed this study and significantly revised this manuscript.
